# Opioid Stewardship and Gastrointestinal (GI)-Sparing Alternatives in Primary Care Pain Management

**DOI:** 10.7759/cureus.86789

**Published:** 2025-06-26

**Authors:** Anusha Khan, Aftab Ahmad, Syed Sharjeel Ibrar, Saira K Awan, Ahmad Hashmat, Alena Berkhamova, Giustino Varrassi

**Affiliations:** 1 Medical Ethics, Northwell Health, Long Island Jewish Medical Center, New York, USA; 2 General Medicine, Cork University Hospital, Cork, IRL; 3 Internal Medicine, Cork University Hospital, Cork, IRL; 4 Neurology, Advanced Neurology P.C., Brooklyn, USA; 5 Medicine, Kabir Medical College, Peshawar, PAK; 6 Nephrology, Richmond University Medical Center, New York, USA; 7 Pain Medicine, Fondazione Paolo Procacci, Rome, ITA

**Keywords:** chronic pain, gastrointestinal risk, non-steroidal anti-inflammatory drugs (nsaids), opioid medication, opioid stewardship

## Abstract

Chronic pain is a widespread condition that affects nearly 30% of adults worldwide and is a leading cause of disability. In the United States, primary care providers (PCPs) play a central role in managing chronic pain. While opioids were historically a common solution, their overuse has contributed to a national crisis involving addiction and misuse. In primary care, managing chronic pain requires balancing effective treatment with minimizing associated risks. Opioid stewardship has emerged as a critical strategy to ensure safe prescribing, while growing awareness of nonsteroidal anti-inflammatory drug (NSAID)-associated gastrointestinal (GI) toxicity underscores the need for GI-sparing alternatives. This narrative review aims to provide PCPs with a comprehensive overview of opioid stewardship principles and evidence-based, GI-sparing alternatives for chronic pain management in outpatient settings. Following SANRA (Scale for the Assessment of Narrative Review Articles) guidelines, a comprehensive literature search was conducted using PubMed, MEDLINE, EMBASE, and the Cochrane Library for English-language publications from 2010 to 2025. Relevant guidelines, reviews, and clinical studies were organized thematically to reflect best practices in opioid risk mitigation, GI risk management, and alternative pain therapies. Key components of opioid stewardship in primary care include patient risk assessment, informed consent, conservative opioid initiation, regular monitoring, and deprescribing when appropriate. GI risk mitigation strategies involve careful NSAID use, co-prescription of proton pump inhibitors (PPIs), and consideration of cyclooxygenase-2 (COX-2) selective agents. Evidence-based, GI-sparing alternatives include acetaminophen, antidepressants, anticonvulsants, topical agents, and non-pharmacological approaches such as physical therapy, cognitive behavioral therapy, and mind-body interventions. The implementation of safe and effective chronic pain management in primary care faces several challenges, including time constraints, limited access to non-opioid or GI-sparing therapies, insurance coverage gaps, and patient resistance to change. This review emphasizes practical strategies such as provider education, interdisciplinary teamwork, and electronic health record (EHR) integration to overcome these barriers. A balanced, patient-centered approach is essential to minimize harm from opioids and GI toxic medications while ensuring long-term, sustainable pain relief.

## Introduction and background

Chronic pain affects approximately 27.5% of adults globally, making it a leading cause of disability [1]. The data are similarly substantial in the United States (US) [2]. Primary care providers (PCPs) are often the first and sometimes only point of contact for patients experiencing chronic pain [3,4]. Historically, opioid medications were widely prescribed to manage moderate to severe pain. However, increased prescribing contributed to a national opioid crisis marked by high rates of misuse, addiction, and overdose deaths [5]. In response, there has been a paradigm shift toward opioid stewardship, an approach that promotes safe, effective, and appropriate use of opioids [6].

Growing awareness of the gastrointestinal (GI) risks linked to commonly used analgesics, especially nonsteroidal anti-inflammatory drugs (NSAIDs), has highlighted the urgent need for safer, GI-sparing alternatives. NSAIDs are well known to cause GI toxicity, including gastritis, peptic ulcer disease, and gastrointestinal bleeding, which can significantly increase morbidity, particularly in older adults and those with existing GI conditions [7]. These adverse effects are largely attributed to the inhibition of cyclooxygenase-1 (COX-1), which not only disrupts the protective lining of the stomach but also suppresses platelet aggregation, thereby increasing the risk of bleeding and ulcer formation [8,9].

Primary care settings are pivotal for implementing opioid stewardship and identifying safe, effective alternatives, including those that minimize GI harm. Given time constraints, limited access to specialists, and patient expectations, PCPs face unique challenges in balancing pain control with both opioid-related and GI-related safety [5,6].

This review outlines the foundational principles of opioid stewardship and discusses GI-sparing, evidence-based alternatives suitable for use in primary care.

## Review

Methodology

This narrative review was conducted in accordance with the SANRA (Scale for the Assessment of Narrative Review Articles) guidelines to ensure methodological rigor and clarity [10]. The SANRA framework guided the planning, literature selection, synthesis, and presentation of the findings related to opioid stewardship and GI-sparing analgesic strategies in primary care.

Aims of the Review

The primary aim of this review is to explore how PCPs can integrate opioid stewardship principles with GI risk mitigation strategies when managing chronic pain. Specifically, this review focuses on evidence-based practices in opioid stewardship applicable to primary care. Furthermore, it identifies pharmacologic and non-pharmacologic alternatives to opioids with a focus on minimizing GI harm. The study also discusses practical implementation strategies and barriers in primary care settings.

Literature Search and Source Selection

A comprehensive search of relevant literature was conducted using the following electronic databases: MEDLINE, PubMed, EMBASE, and Cochrane Library. The search included articles published between 2010 and 2025 in the English language. This ensures contemporary relevance. Key search terms included "opioid stewardship", "chronic pain management", "primary care", "gastrointestinal toxicity", "NSAID alternatives", "gastrointestinal sparing analgesics", and "multimodal pain management". Additional articles were identified through manual searches of reference lists and key guidelines from agencies such as the Centers for Disease Control and Prevention (CDC), World Health Organization (WHO), and American Academy of Family Physicians (AAFP).

Inclusion Criteria

Search was limited to peer-reviewed reviews, clinical guidelines, and original studies relevant to chronic pain, opioid use, and GI safety in primary care, articles addressing adult populations, and literature discussing implementation strategies or challenges in outpatient or primary care settings.

Exclusion Criteria

Non-English language publications, studies focusing exclusively on cancer pain or palliative care, and articles primarily addressing pediatric populations or surgical pain management were excluded.

Critical Appraisal and Synthesis

The sources were critically appraised based on credibility, relevance, and applicability to primary care. Guidelines and systematic reviews were given higher weighting in synthesis due to their methodological rigor. The findings were synthesized thematically to reflect the key principles of opioid stewardship, GI risk mitigation, pharmacologic alternatives, and non-pharmacologic strategies.

This narrative review focuses on clinical applicability in real-world primary care settings and highlights both facilitators and barriers to safe and effective pain management. Conflicting evidence was addressed by discussing the strength and quality of available data while also noting areas of ongoing debate or insufficient research.

Expert and Contextual Consideration

To enhance clinical relevance, the review incorporates expert consensus from national guidelines and integrates perspectives unique to the primary care context, such as system-level constraints, limited specialist access, and patient expectations.

Principles of opioid stewardship

Risk Assessment and Patient Selection

Before initiating opioid therapy, PCPs must assess risk factors for opioid misuse, including personal or family history of substance use disorder, psychiatric comorbidities, and concurrent use of sedatives. Tools such as the Opioid Risk Tool (ORT) [11] and Screening, Brief Intervention, and Referral to Treatment (SBIRT Model) are helpful in guiding decision making [12,13].

In addition to substance misuse risk, assessing a patient’s GI risk factors, such as history of ulcers, *Helicobacter pylori* infection, or concurrent use of anticoagulants or corticosteroids, can inform safer pharmacologic planning when NSAIDs are considered [7,9].

Informed Consent and Treatment Agreements

Patient education about the risks and benefits of opioids is crucial. Treatment agreements or “pain contracts” outline expectations regarding refill policies, safe use, and monitoring procedures. These tools foster shared decision-making and improve accountability. Similarly, discussions should include the GI risks of non-opioid medications, especially NSAIDs. Moreover, the importance of using protective strategies or alternatives when needed [13,14].

Start Low, Go Slow

If opioids are deemed appropriate, they should be initiated at the lowest effective dose with frequent reassessment. Short-acting opioids are preferred initially. Long-acting or high-dose formulations should generally be avoided in opioid-naïve patients (an opioid-naïve patient is new to opioid medicine and their body is not used to it, which makes them more sensitive to the drug’s effects, both good, such as pain relief, and bad, such as slowed breathing) [7,15].

This principle is applied in a similar fashion to NSAIDs. These should also be prescribed at the lowest effective dose for the shortest possible duration. More forethought to be given to patients at risk for GI complications [16]. Co-prescription of proton pump inhibitors (PPIs) or selecting COX-2 selective NSAIDs may offer GI protection but should be individualized [17].

Monitoring and Follow-Up

Routine follow-up visits allow for ongoing assessment of pain, function, side effects, and signs of misuse. Urine drug screening and state prescription drug monitoring programs (PDMPs) can help identify aberrant behavior [5]. Monitoring should also be aimed at keeping an eye out for GI symptoms such as dyspepsia or melena when NSAIDs are used. Early identification of adverse effects may prevent serious complications [18].

Deprescribing and Tapering

Tapering should be considered in the following situations: if opioids are no longer effective, when risks outweigh benefits, or if misuse is suspected. Tapering protocols must be individualized. These should be conducted with patient support, which not only minimizes withdrawal symptoms but also lowers psychological distress [19].

A similar deprescribing approach applies to chronic NSAID use in high-risk patients. This is of particular importance in older adults, where the GI risks may outweigh the benefits. A transition to GI-sparing alternatives can improve safety profiles without compromising effective pain control [15].

Alternatives to opioids in primary care

Non-Opioid Pharmacologic Options

Acetaminophen: Acetaminophen is considered a GI-sparing alternative compared to NSAIDs. It is appropriate for mild to moderate pain and a suitable option for patients who are at an increased risk for GI complications such as ulcers or bleeding. In particular, in conditions like osteoarthritis, long-term pain control is often needed. However, while it has a favorable GI safety profile, acetaminophen must be used cautiously and monitored closely due to hepatotoxicity risk. Therefore, careful daily dose monitoring is essential to avoid and prevent liver damage [20-21].

NSAIDs: These are first line for many types of acute and chronic pain, particularly musculoskeletal and inflammatory conditions [22]. However, their GI toxicity warrants cautious use. GI sparing strategies include using the lowest effective dose, co-prescribing PPIs, or selecting COX-2 inhibitors for patients at increased GI risk [9].

Antidepressants (e.g., TCAs, SNRIs): Antidepressants, such as duloxetine and amitriptyline, are another effective and safe option for managing neuropathic pain, especially in fibromyalgia. These medications work by modulating pain perception through central nervous system pathways, which is independent of inflammation. Duloxetine is a serotonin-norepinephrine reuptake inhibitor (SNRI), while amitriptyline is a tricyclic antidepressant (TCA). Both are frequently used in primary care due to a dual benefit of addressing both chronic pain and associated mood disorders. More importantly, these do not pose GI ulcer risk, making them a valuable GI-sparing alternative [23,24].

Anticonvulsants (e.g., gabapentin, pregabalin): Anticonvulsants, such as gabapentin and pregabalin, are both widely used options in managing neuropathic pain. These are often used for chronic pain-causing conditions, such as postherpetic neuralgia, spinal nerve compression, and diabetic neuropathy. These medications work by modulating calcium channels in the nervous system to reduce the excitability of nerve cells involved in pain signaling. One of their key advantages is that they do not pose a risk of GI bleeding or ulceration, which makes them a safe option for patients who cannot tolerate NSAIDs. However, when prescribed to older adults, these must be used with caution due to their potential to cause sedation, dizziness, and balance impairment. This may lead to an increased risk of falls and cognitive disturbances. Regular monitoring and careful dose titration are recommended to both minimize adverse effects and maximize pain relief [25,26].

Topical agents: Topical agents such as lidocaine patches, capsaicin cream, and topical NSAIDs (e.g., diclofenac gel) offer effective localized pain relief while there is minimal systemic absorption, which significantly reduces the risk of GI and other systemic side effects. These treatments are particularly valuable in managing chronic arthritis-related knee and hand joint pain, musculoskeletal pain, and localized neuropathic pain. Since their action is confined to the site of application, they are ideal for patients with contraindications to oral NSAIDs. These include patients with a history of GI bleeding, peptic ulcers, chronic kidney disease, or cardiovascular disease. Topical therapies are a safer long-term option. Excellent safety profile and ease of use make topical agents an important component of multimodal, GI-sparing pain management strategy [26].

Muscle relaxants and adjuncts: Muscle relaxants, such as cyclobenzaprine, methocarbamol, and tizanidine, can provide short-term relief for acute musculoskeletal pain, especially in cases involving muscle spasms. These medications are often used in the setting of back pain, neck strain, or other soft tissue injuries to help reduce muscle tension and improve comfort. One of their key advantages is that they do not pose a risk of GI bleeding or ulceration, which makes them a safe option for patients who cannot tolerate NSAIDs. However, when prescribed to older adults, these must be used with caution due to their potential to cause sedation, dizziness, and balance impairment. Hence, they are not prescribed for long-term use. Most treatment courses with muscle relaxants are kept at most for two to three weeks [27]. 

In view of the above-mentioned pain management options and principles of opioid stewardship, key GI-sparing strategies are outlined in Table 1. 

**Table 1:** GI-sparing strategies in pain management: a multimodal approach This table outlines key GI sparing strategies to guide safer pain management, particularly when NSAIDs are considered. Strategies include pharmacologic alternatives such as acetaminophen, antidepressants, and topical agents; co-therapies like PPIs; and clinical practices such as risk stratification, dose minimization, patient education, and deprescribing in high-risk populations. These measures aim to reduce GI complications while maintaining effective pain control. GI: gastrointestinal, NSAIDs: nonsteroidal anti-inflammatory drugs, *H. pylori*: *Helicobacter pylori, *e.g.: exempli gratia, PPIs: proton pump inhibitors, COX-2: cyclooxygenase-2 This table was created by the authors. References: [5,7,9,11-27]

Strategy	Description	Examples / notes
Risk stratification	Identify patients with GI risk factors before initiating NSAIDs	History of ulcers, *H. pylori* infection, anticoagulant or corticosteroid use
Alternative medications	Use pain medications that do not pose GI risks	Acetaminophen, antidepressants (e.g., duloxetine), anticonvulsants, and topical agents
Dose and duration minimization	Use the lowest effective dose for the shortest possible time	Applies to both opioids and NSAIDs
Co-prescription of PPIs	Proton pump inhibitors reduce the risk of gastric ulcers from NSAID use	Commonly used in older adults or those with prior GI bleeding
Use of COX-2 selective NSAIDs	Selective NSAIDs have a lower risk of GI toxicity compared to traditional NSAIDs	E.g., celecoxib, but cardiovascular risk must be considered
Topical formulations	Deliver pain relief locally with minimal systemic or GI exposure	Topical diclofenac, lidocaine patches, capsaicin cream
Deprescribing in high-risk patients	Taper or discontinue NSAIDs in older or high-risk patients and switch to safer alternatives	Consider acetaminophen or non-pharmacologic strategies
Patient education	Educate on proper use, risks, and warning signs of GI toxicity	Part of shared decision-making and informed consent
Monitoring for GI symptoms	Regularly check for signs of GI adverse effects during NSAID use	Watch for dyspepsia, melena, anemia, or early signs of GI bleeding

Non-Pharmacologic Approaches

These therapies are inherently GI-sparing and represent essential components of multimodal pain management.

Physical therapy and exercise: Non-pharmacological options such as physical therapy and structured exercise programs have shown promise in managing pain in musculoskeletal conditions, including chronic low back pain, post-injury rehabilitation, and osteoarthritis. These therapies improve joint mobility, strengthen supporting muscles, and enhance overall function. This can reduce pain and improve the patient's quality of life. Personalised exercise plans supervised by trained therapists can not only help prevent further injury but also promote long-term physical activity. In addition, they offer a safe, GI-sparing alternative to pharmacologic therapies [22,28].

Cognitive behavioral therapy (CBT): CBT is a psychological remedy that helps patients channelize negative thoughts and behaviors related to chronic pain. CBT helps improve emotional coping and reduces pain-related anxiety or depression. It has been found to show sustained improvements in pain intensity and functioning. Access to CBT is expanding, especially in primary care and rural settings, with growing digital CBT platforms. Being a GI-safe and non-invasive option, CBT can be a valuable tool in the management of pain [27,28].

Mind-body therapies: These include yoga, mindfulness meditation, tai chi, and progressive muscle relaxation. These techniques can promote the connection between mental and physical health. They work by calming the nervous system, promoting relaxation, and reducing stress. As a result, there is a lower perception of pain. Fibromyalgia, low back pain, and arthritis patients have shown improvement with the use of these therapies. In addition, they are not only non-pharmacologic but also GI-neutral and are hence a good option for patients seeking a safe and complementary alternative for pain management [27-29].

Manual therapies: Osteopathic manipulative treatment, chiropractic adjustments, and therapeutic massage can be beneficial in relieving musculoskeletal pain through soft tissue mobilization, realignment, and improved circulation. In a selected patient population with muscle tightness, mechanical low back pain, and neck pain, these treatment therapies could be applied. Manual therapies are also generally a good and safer alternative for pain management and do not carry GI-related risks [27,28,30].

Acupuncture: Acupuncture is a traditional Chinese medicine technique that involves the insertion of small, fine needles in specific body points that promote healing and modulate pain pathways. Although acupuncture is considered safe when performed by trained professionals, it has shown only moderate pain relief. With minimum systemic side effects or GI complications, acupuncture is an excellent complementary therapy for patients who cannot tolerate NSAIDs or opioids [27,28,31].

The various non-opioid pharmacologic and non-pharmacologic options, along with their indications, GI-sparing benefits, and clinical considerations, are outlined in Table 2. These strategies support a multimodal approach to pain management aimed at minimizing opioid reliance and reducing gastrointestinal risk.

**Table 2:** Alternatives to opioids in primary care This table outlines a range of non-opioid pharmacologic and non-pharmacologic treatments used in primary care settings for the management of pain. It highlights the indications, benefits, and clinical considerations associated with each option, with a focus on GI-sparing properties. Non-opioid pharmacologic options include acetaminophen, NSAIDs, antidepressants, anticonvulsants, topical agents, and muscle relaxants. Non-pharmacologic approaches include physical therapy, CBT, mind-body therapies, manual therapies, and acupuncture. These multimodal strategies are designed to enhance pain control while minimizing opioid use and reducing GI-related adverse effects. GI: gastrointestinal, NSAIDs: nonsteroidal anti-inflammatory drugs, COX-2: cyclooxygenase-2, PPIs: proton pump inhibitors, TCAs: tricyclic antidepressants, SNRIs: serotonin norepinephrine reuptake inhibitors, BP: blood pressure, CBT: cognitive behavioral therapy This table was created by the authors. References: [9,20-31]

Category	Treatment option	Indications	Benefits	Cautions/considerations
Non-opioid pharmacologic options	Acetaminophen	Mild to moderate pain, osteoarthritis	GI sparing, widely available	Risk of hepatotoxicity with high doses
	NSAIDs	Musculoskeletal pain, inflammatory conditions	First line for many types of pain	GI toxicity; consider COX-2 inhibitors, PPIs, low dose
	Antidepressants (e.g., TCAs, SNRIs)	Neuropathic pain, fibromyalgia	No GI ulcer risk, effective for chronic pain	Anticholinergic effects (TCAs), BP changes (SNRIs)
	Anticonvulsants (gabapentin, pregabalin)	Neuropathic pain	No GI bleeding risk, effective for nerve pain	Sedation, dizziness, caution in the elderly
	Topical agents (lidocaine, capsaicin, topical NSAIDs)	Localized pain (e.g., joint, nerve pain)	Minimal systemic absorption, GI sparing	Skin irritation is possible, limited to localized pain areas
	Muscle relaxants	Acute musculoskeletal pain	Short-term relief, non-GI toxic	Sedation, not recommended for long-term use
Non-pharmacologic approaches	Physical therapy and exercise	Osteoarthritis, chronic low back pain	Improves function and pain, with no systemic side effects	Requires patient participation and adherence
	CBT	Chronic pain coping, fibromyalgia	Reduces distress, available digitally	Access and motivation may vary
	Mind-body therapies (e.g., yoga, meditation)	Chronic pain, stress-related conditions	Safe, improves well-being and function	Evidence varies; patient willingness is needed
	Manual therapies (massage, manipulation)	Select musculoskeletal conditions	Non-invasive, beneficial for some	Variable provider availability and insurance coverage
	Acupuncture	Chronic low back pain, knee OA	Moderate evidence of benefit, no GI side effects	Access, cost, and response vary by individual

Implementation strategies in primary care

Education and Training

Ongoing training for PCPs on pain management guidelines, opioid prescribing laws, and GI risk mitigation strategies is essential to ensure they are well informed about the recent advances and strategies to reduce GI risks. Continuing medical education (CME) programs can be vital in offering learning opportunities accessible to clinicians. In addition, clinical tools, including prescribing checklists, and dosing calculators, can support safer prescribing. Such training approaches can improve physician confidence and competence in managing chronic pain safely while reducing the risk of adverse effects like GI bleeding [32-33].

Interdisciplinary Care

Collaborating with pharmacists, physical therapists, behavioral health specialists, and pain specialists enhances care quality and facilitates the development of comprehensive and individualized treatment plans. This can ensure access to safer and GI-sparing options. A collaborative model can enhance care quality, improve patient outcomes, and expand access to safer alternatives [34-35].

Workflow Integration

Embedding risk assessment tools, PDMP access, and prescribing guidance, including GI risk considerations, into electronic health records (EHRs) streamlines safer practices. For instance, embedding risk assessment tools helps clinicians identify patients who may be at higher risk for opioid misuse or GI complications. Access to PDMPs within the EHR would allow for quick checks on a patient’s prescription history. This can improve oversight and reduce the risk of duplicate or dangerous prescriptions, along with guiding GI sparing and safer options [18,28].

Patient Engagement

Educating patients about the chronic nature of pain and setting realistic expectations, along with discussing the risks of GI complications from certain medications, can improve adherence and satisfaction. When patients understand the risks and benefits of different treatments, they are not only more likely to adhere to prescribed regimens but also take an informed decision in considering alternative therapies, such as behavioral interventions or physical therapy [32,36].

Challenges and barriers

Challenges include time constraints during visits, which can limit comprehensive assessment. Limited access to non-opioid and GI-sparing therapies in rural or underserved areas is another barrier to ensuring safer pain management options. Stigma and fear around opioid tapering or unfamiliar therapies may result in reduced patient cooperation. Insurance limitations often restrict coverage for integrative and behavioral approaches that are gastroprotective in nature. Addressing these barriers requires policy advocacy, systemic change, and structured resource allocation to ensure equitable access to both opioid stewardship and GI-safe alternatives. In addition, using technology such as telehealth platforms, artificial intelligence (AI) assisted clinical tools, and virtual pain management clinics can streamline care coordination, expand access, and support personalized treatment plans in both urban and remote settings [37-39].

Limitations

There are a few limitations to this study. As a narrative review, this article is subject to selection and interpretation bias. We only included articles published in the English language, while studies published in other languages were excluded. In addition, the synthesis reflects current knowledge and may require updates as new evidence emerges.

## Conclusions

PCPs play a pivotal role in integrating opioid stewardship with GI safety in pain management. Managing pain requires a balanced approach that prioritizes both safety and efficacy. A multimodal, individualized approach supported by judicious prescribing, education, and interdisciplinary collaboration can ensure better outcomes while minimizing harm. Embedding these practices into clinical workflows via EHRs, decision support tools, and accessible CME programs enhances safety and consistency. Addressing implementation barriers requires individual provider efforts along with broader systemic support, including policy reform and technology integration. In conclusion, embracing the principles of opioid stewardship and prioritizing GI safety, primary care teams can provide more effective, safer, and sustainable care for patients.
